# The reliability of a newborn foot length measurement tool used by community volunteers to identify low birth weight or premature babies born at home in southern Tanzania

**DOI:** 10.1186/1471-2458-14-859

**Published:** 2014-08-20

**Authors:** Tanya Marchant, Suzanne Penfold, Elibariki Mkumbo, Donat Shamba, Jennie Jaribu, Fatuma Manzi, Joanna Schellenberg

**Affiliations:** Faculty of Infectious and Tropical Disease, London School of Hygiene and Tropical Medicine, Keppel St, London, WC1E 7HT UK; MARCH (Maternal, Adolescent, Reproductive and Child Health Centre), London School of Hygiene and Tropical Medicine, Keppel Street, London, WC1E 7HT UK; Ifakara Health Institute, Dar es Salaam, P.O. BOX 78373, Tanzania

## Abstract

**Background:**

Low birthweight babies need extra care, and families need to know whether their newborn is low birthweight in settings where many births are at home and weighing scales are largely absent. In the context of a trial to improve newborn health in southern Tanzania, a counselling card was developed that incorporated a newborn foot length measurement tool to screen newborns for low birth weight and prematurity. This was used by community volunteers at home visits and shows a scale picture of a newborn foot with markers for a ‘short foot’ (<8 cm). The tool built on previous hospital based research that found newborn foot length <8 cm to have sensitivity and specificity to identify low birthweight (<2500 g) of 87% and 60% respectively.

**Methods:**

Reliability of the tool used by community volunteers to identify newborns with short feet was tested. Between July-December 2010 a researcher accompanied volunteers to the homes of babies younger than seven days and conducted paired measures of newborn foot length using the counselling card tool and using a plastic ruler. Intra-method reliability of foot length measures was assessed using kappa scores, and differences between measurers were analysed using Bland and Altman plots.

**Results:**

142 paired measures were conducted. The kappa statistic for the foot length tool to classify newborns as having small feet indicated that it was moderately reliable when applied by volunteers, with a kappa score of 0.53 (95% confidence interval 0.40 – 0.66) . Examination of differences revealed that community volunteers systematically underestimated the length of newborn feet compared to the researcher (mean difference −0.26 cm (95% confidence interval −0.31—0.22), thus overestimating the number of newborns needing extra care.

**Conclusions:**

The newborn foot length tool used by community volunteers to identify small babies born at home was moderately reliable in southern Tanzania where a large number of births occur at home and scales are not available. Newborn foot length is not the best anthropometric proxy for birthweight but was simple to implement at home in the first days of life when the risk of newborn death is highest.

## Background

In 2012, the identification of low birth weight infants (<2500 g) within 48 hours of birth among those born at home was ranked by scientists and public health practitioners as the number one research priority to reduce global mortality from preterm birth and low birth weight [1]. Low birth weight infants need additional care: each year in sub-Saharan Africa an estimated 1.2 million newborns die during the first month of life [2]. Being born too small – because of low birth weight, prematurity, or intra-uterine growth retardation – is a very strong predictor of newborn mortality [3, 4]. Life saving interventions [5] implemented in the community and in health facilities could save as many as 85% of African maternal, newborn and child deaths if optimal coverage was achieved, [6] but despite some improvements, intervention coverage still remains low for the majority of families across Africa [7].

One immediate obstacle to realising the full potential to save lives is that, despite a global increase in skilled attendance at birth in the last decade, around half of all births in Africa continue to occur at home, [8, 9] and most families have no contact with formal health services during the first week of a newborn’s life. In such settings, community health workers or volunteers have considerable potential to improve newborn survival by promoting skin-to-skin contact, thermal care, immediate and frequent breastfeeding, and active care seeking [10]. A meta-analysis of studies to evaluate the impact of home visits to newborns in programmatic settings estimated a 12% reduction in newborn mortality, equivalent to a five percent reduction in overall child mortality [11].

One persistent limitation of home visits is that community members, including volunteers, usually lack the tools and measurement skills with which to identify newborns as small and at-risk. We previously reported a hospital based study showing that short foot length had the potential to be used as a low-cost tool to identify low birth weight or preterm newborns born at home in the event that weighing scales and clinical assessment were not available [12].

Here we report on a community based study to estimate the reliability of a simple foot length measurement tool used by community volunteers to identify low birth weight or premature babies born at home in the first days of life.

## Methods

### Setting

The INSIST project (Improving Newborn Survival in Southern Tanzania, clinical trials identifier NCT01022788) aimed to develop, implement and evaluate a community based package for improved newborn care, focussing on home-based counselling visits by community volunteers to women in pregnancy and the early neonatal period. The community-based package was implemented in half of all wards in six districts of Lindi and Mtwara Regions which are characterised as rural with a predominantly unpaved road network. The most common occupations are subsistence farming and small scale trading, and neonatal and infant mortality are high [13, 14]. Neonatal and infant mortality in the area are high (estimated to be 43/1000 and 76/1000 live births respectively) [13]. Around half of babies are born in a health facility, [15] and the public health system offers a varying quality of care [13, 14].

**Figure 1:**
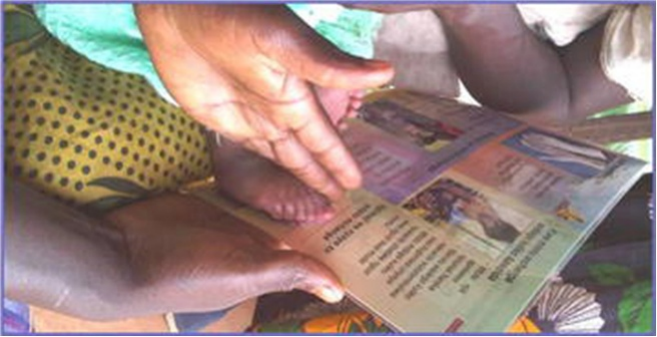
**Using the counselling card to categorise newborn foot length as “very short” (<7 cm), “short” (7–7.9 cm) or “not short” (8 + cm) in Mtwara Region, Tanzania (here showing a newborn with “not short” foot length).**

The INSIST project developed a counselling card for community volunteers to use when promoting healthy newborn behaviours in the home (Figure 1). Families are given their own copy of this card, and community volunteers have a laminated version. To aid community identification of low birth weight or preterm newborns, the card also shows a scale picture of a newborn foot showing cut-offs for very short (<7 cm) and short (7.0-7.9 cm) feet, and not short (8.0 + cm) . These categories of foot length were reported to be indicative of low birth weight or prematurity [12]. It is this measurement tool that is evaluated here.

### Study design and participants

This was a cross sectional study of paired observations of newborn foot length, using the laminated counselling card, with one observation in each pair being done by a project researcher and the second by the community volunteers. In addition, the researcher and volunteers also measured foot length using a plastic ruler. Based on previous research [12] 50% of newborns were expected to have a foot length shorter than 8 cm (a proxy for birth weight less than or more than 2500 g). 143 observation pairs would be sufficient to estimate an expected kappa of 0.4 and its 95% confidence interval (indicating fair agreement between different observers using the same measurement tool) for rating newborns as having feet shorter or longer than 8 cm.

The community volunteers attend quarterly review meetings to share results, review their experience of counselling families, and plan for the next quarter. Between July and December 2010, volunteers attending these review meetings in all six districts of the INSIST project were asked to identify any newborns in their villages who were less than 7 days old at the time of the meeting. Within 24 hours of each review meeting a joint home visit was then made to these infants by a member of the research staff (EM) and the individual community volunteer.

At the home visit, the age of the newborn was confirmed, and consent to proceed sought from the caregiver, with the exception of those newborns who were over seven days of age on the day of visit, or who showed signs of distress. For consenting newborns, a standard operating procedure was followed to measure newborn foot length. First, the community volunteer categorised the newborn foot length using the laminated counselling card by placing the right foot against the printed picture and reading whether length was above or below labelled measurement lines), then recorded the newborn foot length (right foot) in centimetres (to one decimal place) using a hard transparent plastic ruler. Finally, the researcher repeated both measures. Each newborn was assigned a unique identification number and all measures were conducted independently and findings recorded on separate forms.

### Training and quality control

Immediately prior to the start of data collection, the researcher spent two consecutive days at Ligula hospital, together with a paediatrician, each day measuring the foot length of a minimum of ten newborns using both the counselling card and the ruler. Community volunteers received an explanation about how to measure newborn foot length as part of their initial 5-day training by INSIST, were provided with written instructions about how to use the counselling card, and had the opportunity to ask questions about its implementation during quarterly review meetings. One in ten home visits was accompanied by a second researcher (JJ) to check that standard procedures were being followed.

### Data processing and statistical analysis

Data were recorded on paper forms at the newborn’s house and within 48 hours forms were returned to the project office where they were double entered and reconciled in Epi-info.

Characteristics of the newborns, their mothers, and the community volunteers were summarised, and the foot length outcomes derived from the ruler and the counselling card calculated. The intra-method reliability of the counselling card used by the volunteers or the researcher to detect small babies was estimated using kappa statistics [16]. Foot length measures were summarised, and the mean difference and standard deviation calculated. The extent of agreement between the two measurers was further examined by generating Bland and Altman plots [17] which plot the differences between pairs of measurement against the mean of each pair of measures.

### Ethical approval

The study was approved by the Institutional Review Board of Ifakara Health Institute, Tanzania, the Medical Research Coordinating Committee, Tanzania, and the London School of Hygiene and Tropical Medicine, UK. Written, informed consent was obtained from the caregiver of each infant in the study.

## Results

### Study population

Between 21 July 2010 and 7 Jan 2011 the researcher accompanied 119 community volunteers (median age 30 years) to the homes of 144 mothers (median age 26 years) and their newborns (median age 4 days, range 1–7), from across all 6 districts of the project. Complete data were recorded for 142 newborns and are analysed here. All volunteers had completed primary education, and 27% (32/119) had attended some secondary schooling.

### Categorising babies as small using the counselling card

Forty six percent (95% CI 38–55, n = 66/142) of newborns were categorised as having short foot length (<8 cm) by the researcher, and 59% (95% CI 51–67, n = 84 of 142) by the community volunteer (Table 1).

**Table 1:** **Newborn foot lengths measured by community volunteers and researcher using a hard transparent plastic ruler and using the counselling card**

	Community volunteer N = 142	Researcher N = 142
Mean length in cm (SD)	7.8 (0.3)	8.1 (0.3)
*Range (cm)*	*7.0 - 8.7*	*7.2 – 8.8*
	n,% (95% confidence interval)	n,% (95% confidence interval)
Ruler measures categorised:		
<7 cm	0	0
7.0-7.9 cm	77, 54% (46–63)	49, 35% (27–43)
≥8 cm	65, 46% (37–54)	93, 65% (57–73)
Counselling card categories:		
<7 cm	4, 3% (0–7)	1, 1% (0–4)
7.0-7.9 cm	80, 56% (48–65)	65, 46% (37–54)
≥8 cm	58, 41% (33–49)	76, 54% (45–62)

### Reliability of the counselling card tool

The kappa statistic for agreement between the researcher and volunteer assessments using the counselling card was 0.53 (95% confidence interval 0.40-0.66; expected agreement 49%, actual agreement 76%), a value usually interpreted as reflecting moderate agreement [16].

### Pattern of difference between measurers

**Figure 2:**
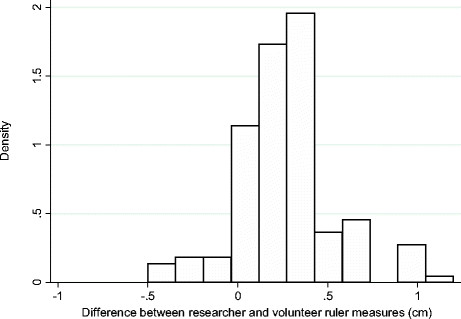
**Distribution of differences between researcher and community volunteer ruler measures of 142 newborn feet (cm).**

**Figure 3:**
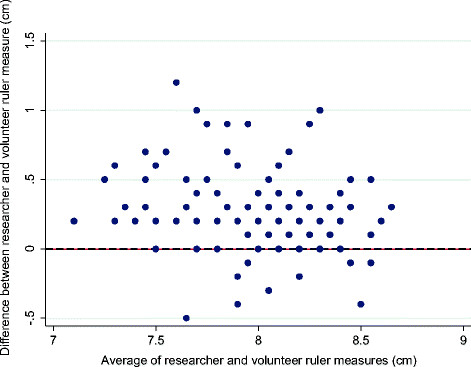
**Bland and Altman plot showing differences between researcher and community volunteer ruler measurements of 142 newborn feet against the average of these two measures (cm).**

The pattern of difference was examined in more detail using the plastic ruler foot length estimates, recorded in centimetres. On average, the researcher recorded longer foot lengths than did the community volunteers (Table 1). The mean foot length recorded by the researcher was 0.26 cm longer that that recorded by the volunteers (SD 0.27, 95% confidence interval of difference between the means 0.22-0.30). There was a positive skew in the distribution of difference between researcher and volunteer measures (Figure 2), and after plotting the difference against the average of paired measures no evidence of bias for different sized feet was observed (Figure 3).

## Discussion

In this community based study we have shown that a newborn foot length measurement tool, printed on a counselling card, can be used at the community level with moderate reliability by community volunteers who have received 5 days of training in total, which included approximately 5 hours on foot size measurement. This training was supplemented by quarterly review meetings, lasting a few hours each time, at which volunteers were encouraged to discuss their experiences from the field.

Qualitative and quantitative evidence from the study area [15, 18] informed the healthy newborn behaviours to be promoted by INSIST volunteers during the main INSIST trial. The key behaviours were hygiene during childbirth, immediate and exclusive breastfeeding, and extra care, including skin to skin care, for low birth weight or premature babies. In order to identify low birth weight or premature newborns, volunteers required a simple and easily sustainable identification tool. Key stakeholders at national level advised that weighing scales would not be sustainable, and many primary health care facilities do not have scales, thus the foot length tool was developed [19]. Our findings show that community volunteers tended to under estimate newborn foot length. The implication of this may have been that a large number of newborns were said to be in need of extra care, something that could have led volunteers and parents to lose confidence in the identification tool. Nonetheless, by putting focus on examining the newborn, the tool may have provided a starting point for discussion with parents about whether or not they thought that their baby needed extra care – beyond that recommended for every newborn - in the first days of life. The issue of early care seeking from formal health providers urgently needs to be addressed. A meta-analysis of care seeking for neonatal illness in low and middle income countries [20] highlighted that there is a paucity of high quality data on the subject, especially from Africa, and estimated that just 20% of caregivers of sick newborns reported seeking care from a health care provider outside the home. In southern Tanzania, previous studies have suggested that care seeking is particularly low for babies born at home [14] – precisely the group of newborns that were targeted by the foot length tool.

The goal of health care providers is to have contact with every newborn at birth (through facility delivery) or immediately after (through post-natal care) and take a high quality measure of birth weight to inform decision making about any care needed. But sub-optimal coverage of institutional deliveries and post-natal care means that around half the newborns of sub-Saharan Africa do not have this opportunity and alternative methods to identify small babies should be encouraged if they can be linked to identification of newborn danger signs. In an analysis of Demographic and Health Survey data from three countries, [21] mother’s perception of a baby’s size at birth was found to be closely linked to birth weight at national levels, although within communities there was considerable variation in size perceptions. The authors concluded that care was needed when interpreting individual maternal perceptions because of the influence of other variables such as gender of the child, place of birth, and the size of other babies in the local community.

Measuring newborn foot length has been found to be acceptable, [12] but of the anthropometric proxies for low birth weight it is not the most accurate. Our earlier hospital based study estimated the sensitivity and specificity of foot length <8 cm to predict low birth weight (<2500 g) to be 0.87 and 0.60 respectively [12]. A meta-analysis of evidence from hospital studies concluded that chest circumference had the highest accuracy when predicting low birth weight, compared to other anthropometric measures, [22] but its main limitation for application in a community setting was that it can be difficult to measure accurately without medical training, and can be disruptive to the newborn since it requires undressing [23, 24]. Further to this, integrating the foot length tool as a printed image on a counselling card may not have been the most accurate method of determining foot length, and some discordant categorisation was observed between the counselling card and the ruler. But having just one simple, cheap, locally produced tool for volunteers to carry and explain, and being able to leave a copy of this tool with families, may have outweighed this limitation by removing important barriers to its use.

## Conclusions

A simple newborn foot length measurement tool can be applied by community volunteers with moderate reliability. However, using this tool volunteers over estimated the number of small newborns needing extra care by thirteen percent compared to estimates by the project researcher. Newborn foot length is simple to understand, easy to construct and can be implemented at home in the first days of life when the risk of newborn death is highest, but monitoring its use and providing supportive supervision to users of the tool is important.

## References

[1] Bahl R, Martines J, Bhandari N, Biloglav Z, Edmond K, Iyengar S, Kramer M, Lawn JE, Manandhar DS, Mori R, Rasmussen KM, Sachdev HP, Singhal N, Tomlinson M, Victora C, Williams AF, Chan KY, Rudan I (2012). Setting research priorities to reduce global mortality from preterm birth and low birth weight by 2015. J Global Health.

[2] Lozano R, Wang H, Foreman KJ, Rajaratnam JK, Naghavi M, Marcus JR, Dwyer-Lindgren L, Lofgren KT, Phillips D, Atkinson C, Lopez AD, Murray CJ (2011). Progress towards Millennium Development Goals 4 and 5 on maternal and child mortality: an updated systematic analysis. Lancet.

[3] UNICEF WHO The World Bank UNFPA: *Levels & Trends in Child Mortality, Report 2012*. New York: UNICEF 2012; http://www.unicef.org/videoaudio/PDFs/UNICEF_2012_child_mortality_for_web_0904.pdf

[4] Marchant T, Willey B, Katz J, Clarke S, Kariuki S, ter Kuile F, Lusingu J, Ndyomugyenyi R, Schmiegelow C, Watson-Jones D, Armstrong Schellenberg J (2012). Neonatal mortality risk associated with preterm birth in East Africa, adjusted by weight for gestational age: individual participant level meta-analysis. PLoS Med.

[5] Partnership for Maternal Newborn & Child Health (2011). Child Health: Essential Interventions, Commodities and Guidelines for Reproductive, Maternal, Newborn and Child Health. A Global Review of the Key Interventions Related to Reproductive, Maternal Newborn and Child Health (RMNCH).

[6] Friberg IK, Kinney MV, Lawn JE, Kerber KJ, Odubanjo MO, Bergh AM, Walker N, Weissman E, Chopra M, Black RE, Axelson H, Cohen B, Coovadia H, Diab R, Nkrumah F, Science in Action: Saving the lives of Africa's Mothers, Newborns, and Children working group (2010). Sub-Saharan Africa’s mothers, newborns, and children: how many lives could be saved with targeted health interventions?. PLoS Med.

[7] Bryce J, Daelmans B, Dwivedi A, Fauveau V, Lawn JE, Mason E, Newby H, Shankar A, Starrs A, Wardlaw T (2008). Countdown to 2015 for maternal, newborn, and child survival: the 2008 report on tracking coverage of interventions. Lancet.

[8] WHO UNICEF UNFPA and The World Bank: **Trends in maternal mortality:1990 to 2010.** http://whqlibdocwhoint/publications/2012/9789241503631_engpdf 2012 (Last accessed 3rd August 2013)

[9] Save the Children: **State of the World's Mothers.** http://wwwsavethechildrenweborg/SOWM-2013/ 2013 (Last accessed 3 August 2013)

[10] WHO and UNICEF: **Home visits for the newborn child: a strategy to improve survival: Joint statement.** http://whqlibdocwhoint/hq/2009/WHO_FCH_CAH_0902_engpdf 2009 24809117

[11] Kirkwood BR, Manu A, ten Asbroek AH, Soremekun S, Weobong B, Gyan T, Danso S, Amenga-Etego S, Tawiah-Agyemang C, Owusu-Agyei S, Hill Z (2013). Effect of the Newhints home-visits intervention on neonatal mortality rate and care practices in Ghana: a cluster randomised controlled trial. Lancet.

[12] Marchant T, Jaribu J, Penfold S, Tanner M, Armstrong Schellenberg J (2010). Measuring newborn foot length to identify small babies in need of extra care: a cross sectional hospital based study with community follow-up in Tanzania. BMC Public Health.

[13] Armstrong Schellenberg JR, Mrisho M, Manzi F, Shirima K, Mbuya C, Mushi AK, Ketende SC, Alonso PL, Mshinda H, Tanner M, Schellenberg D (2008). Health and survival of young children in southern Tanzania. BMC Public Health.

[14] Mrisho M, Schellenberg D, Manzi F, Tanner M, Mshinda H, Shirima K, Msambichaka B, Abdulla S, Schellenberg JA (2012). Neonatal deaths in rural southern Tanzania: care-seeking and causes of death. ISRN Pediatrics.

[15] Penfold S, Hill Z, Mrisho M, Manzi F, Tanner M, Mshinda H, Schellenberg D, Armstrong Schellenberg JR (2010). A large cross-sectional community-based study of newborn care practices in southern Tanzania. PLoS One.

[16] Sim J, Wright CC (2005). The kappa statistic in reliability studies: use, interpretation, and sample size requirements. Phys Ther.

[17] Bland JM, Altman DG (1995). Comparing methods of measurement: why plotting difference against standard method is misleading. Lancet.

[18] Shamba DD, Schellenberg J, Penfold SC, Mashasi I, Mrisho M, Manzi F, Marchant T, Tanner M, Mshinda H, Schellenberg D, Hill Z (2013). Clean home-delivery in rural Southern Tanzania: barriers, influencers, and facilitators. J Health Popul Nutr.

[19] Penfold S, Shamba D, Hanson C, Jaribu J, Manzi F, Marchant T, Tanner M, Ramsey K, Schellenberg D, Schellenberg JA (2013). Staff experiences of providing maternity services in rural southern Tanzania - a focus on equipment, drug and supply issues. BMC Health Serv Res.

[20] Herbert HK, Lee AC, Chandran A, Rudan I, Baqui AH (2012). Care seeking for neonatal illness in low- and middle-income countries: a systematic review. PLoS Med.

[21] Channon AA (2011). Can mothers judge the size of their newborn? Assessing the determinants of a mother’s perception of a baby’s size at birth. J Biosoc Sci.

[22] Goto E (2011). Meta-analysis to estimate the correlation coefficients between birthweight and other anthropometric measurements at birth. Indian J Pediatr.

[23] Mullany LC, Darmstadt GL, Khatry SK, Leclerq SC, Tielsch JM (2007). Relationship between the surrogate anthropometric measures, foot length and chest circumference and birth weight among newborns of Sarlahi, Nepal. Eur J Clin Nutr.

[24] James DK, Dryburgh EH, Chiswick ML (1979). Foot length–a new and potentially useful measurement in the neonate. Arch Dis Child.

[CR25] The pre-publication history for this paper can be accessed here:http://www.biomedcentral.com/1471-2458/14/859/prepub

